# Vertebral remodeling with denosumab after infection control in *Escherichia coli* spondylodiscitis: case report

**DOI:** 10.1093/jbmrpl/ziag040

**Published:** 2026-03-16

**Authors:** Bing-Hao Lu, Tung-Ying Lin, Kuo-Yuan Huang

**Affiliations:** Department of Education Center, National Cheng Kung University Hospital, Tainan 70403, Taiwan; Department of Medical Education and Research, Kaohsiung Veterans General Hospital, Kaohsiung 813414, Taiwan; Department of Orthopedics, National Cheng Kung University Hospital, College of Medicine, National Cheng Kung University, Tainan 70403, Taiwan

**Keywords:** infectious spondylodiscitis, *Escherichia coli* bacteremia, osteoclastogenesis, denosumab, vertebral remodeling, adjunct therapy

## Abstract

Infectious spondylodiscitis can cause progressive vertebral destruction and instability even after infection control, and some patients decline or are unsuitable for surgery. We report a 67-yr-old man with poorly controlled type 2 diabetes mellitus and hypertension who developed *Escherichia coli* bacteremia and subsequent infectious spondylodiscitis. Four months after treatment of a traumatic open tibial wound with debridement and split-thickness skin grafting, he presented with progressive low back pain, intermittent fever, and systemic illness with acute kidney injury and hepatic dysfunction. A distal common bile duct stone was treated endoscopically, but fever persisted. A gallium-67 scan raised suspicion for L2-L3 involvement, and LS MRI demonstrated L2-L3 infectious spondylodiscitis with extensive paraspinal and epidural inflammatory changes and bilateral psoas abscesses. CT-guided aspiration and bone biopsy were culture-negative. He improved clinically after approximately 5 wk of antimicrobial therapy and declined operative debridement or stabilization. One week after completing antibiotics, denosumab (60 mg, s.c.) was administered. Given that denosumab is not a guideline-supported therapy for infectious spondylodiscitis, it was used off-label as a speculative adjunct to suppress inflammation-associated bone resorption; a second dose was given 6 mo later. Back pain and function improved with bracing, and serial radiographs showed new bone formation along the superior endplate of L3 with progressive remodeling. At 6-yr follow-up, the patient remained free of adverse effects and demonstrated spontaneous fusion of L2 and L3 with sustained symptom relief. This report is descriptive and hypothesis-generating. The observed remodeling may also reflect expected post-infectious healing under conservative management. Nevertheless, the course raises a testable hypothesis that carefully timed antiresorptive therapy after confirmed infection control may support structural recovery in selected nonoperative patients with marked infection-related vertebral osteolysis.

## Graphical Abstract

**Graphical Abstract:**
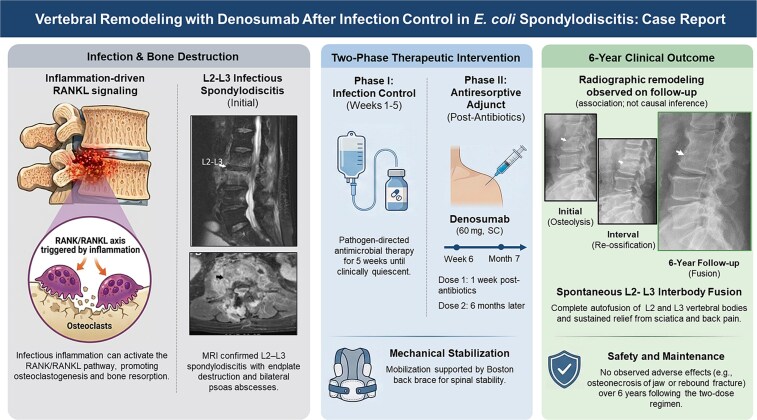

## Introduction

Infectious spondylodiscitis is a serious infection of the intervertebral disc and adjacent vertebral endplates and bodies that can lead to progressive osteolysis, deformity, neurologic compromise, and mechanical instability. Clinical presentation is often nonspecific, with back pain and systemic symptoms variably accompanied by fever and neurologic deficits. Infection typically arises from hematogenous seeding, direct inoculation, or contiguous spread from adjacent foci, including urinary tract or postoperative sources.^1^

Prolonged, pathogen-directed antimicrobial therapy remains the cornerstone of management, whereas surgical debridement and stabilization are reserved for selected patients with refractory infection, progressive instability, large abscess burden, or neurologic deterioration.^2^ Even when infection is eradicated, however, antimicrobial therapy does not directly address the downstream biology of infection-associated bone loss. Consequently, patients with substantial endplate destruction may remain at risk for vertebral collapse, epidural extension, and persistent pain despite microbiologic control.

From a bone biology perspective, infection-associated inflammation can amplify osteoclastogenesis through the RANK/RANKL axis and related downstream signaling, thereby accelerating vertebral bone loss.^3^ Experimental infection models further support this concept, showing that infection induces robust RANKL expression and that RANKL blockade can prevent infection-associated bone loss.^4^ This raises the possibility that, once the microbial burden is clinically controlled, targeted suppression of osteoclast-mediated resorption could help to limit further structural deterioration and allow reparative remodeling to proceed.

Denosumab is a fully human monoclonal antibody against RANKL that potently inhibits osteoclast differentiation and survival and has established antiresorptive efficacy in osteoporosis and other high-risk osteolytic conditions, including malignancy-related bone disease and giant cell tumor of bone.^5–8^ Despite this biological rationale, clinical experience with denosumab as an adjunct after infection control in infectious spondylodiscitis remains limited, and long-term radiographic outcomes are seldom reported.

Here, we report a case of infectious spondylodiscitis complicated by *Escherichia coli* bacteremia in which adjunctive denosumab was initiated after completion of antibiotic therapy, followed by sustained clinical improvement and radiographic evidence of vertebral remodeling on long-term follow-up, and we discuss timing and safety considerations for this approach.

### Case

A 67-yr-old male with a history of poorly controlled type 2 diabetes mellitus and hypertension presented to our hospital with progressive soft-tissue necrosis of the left lower leg. He had sustained a traumatic open wound over his left tibia in a motor vehicle accident approximately 1 mo prior and underwent local debridement at a local clinic. Due to persistent wound discharge and failure to heal, he was admitted to our hospital and underwent wound debridement and split-thickness skin graft. The postoperative course was uneventful, and the patient was discharged with adequate graft take.

Four months later, he developed progressive low back pain, accompanied by fatigue, nausea, altered mental status, and intermittent fever, which prompted admission to the emergency department. Initial laboratory tests revealed metabolic acidosis, acute kidney injury (serum creatinine: 5.62 mg/dL), hyperbilirubinemia, and hyperglycemia (HbA1c: 12.6%). Brain CT showed no evidence of intracranial hemorrhage. Blood cultures grew *E coli*, and i.v. antibiotic therapy was initiated with ceftriaxone and subsequently switched to cefepime.

**Figure 1:**
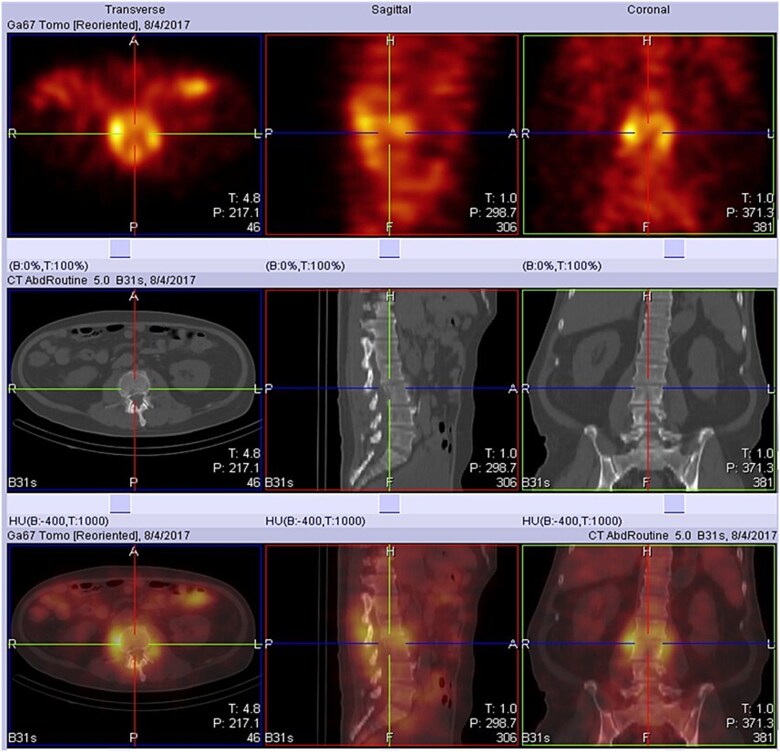
Gallium-67 SPECT/CT demonstrates focally increased radiotracer uptake at the L2 to L3 intervertebral disc space with associated uptake in the adjacent paraspinal soft tissues, consistent with active infectious spondylodiscitis.

**Figure 2:**
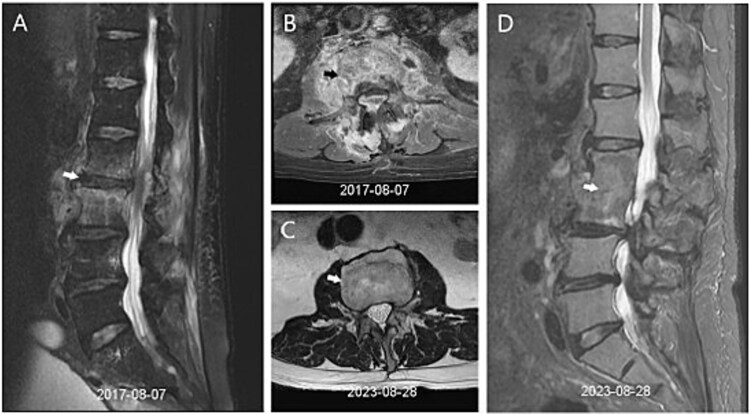
MRI findings of L2–L3 infectious spondylodiscitis and subsequent structural recovery. (A, B) Initial MRI demonstrates L2–L3 spondylodiscitis with paraspinal and epidural involvement and bilateral psoas abscesses. (C, D) Follow-up imaging shows interval improvement after treatment, with subsequent vertebral bone regeneration and structural restoration.

Because fever and elevated liver enzymes persisted, magnetic resonance cholangiopancreatography was performed, revealing a distal common bile duct stone. He subsequently underwent endoscopic retrograde cholangiopancreatography with sphincterotomy, balloon dilatation, and stone extraction. Despite biliary decompression, intermittent fever continued, and antimicrobial therapy was broadened to piperacillin/tazobactam. Gallium-67 imaging raised suspicion for L2-L3 infectious spondylodiscitis (Figure 1). MRI of the lumbosacral spine confirmed infectious spondylodiscitis at the L2-L3 level, accompanied by extensive paraspinal and epidural involvement and bilateral psoas abscesses (Figure 2). CT-guided needle aspiration with bone biopsy was performed but yielded no bacterial growth. After resolution of fever and overall clinical stabilization, antibiotic therapy was de-escalated to oral cefixime. The total duration of antimicrobial treatment was approximately 5 wk.

**Figure 3:**
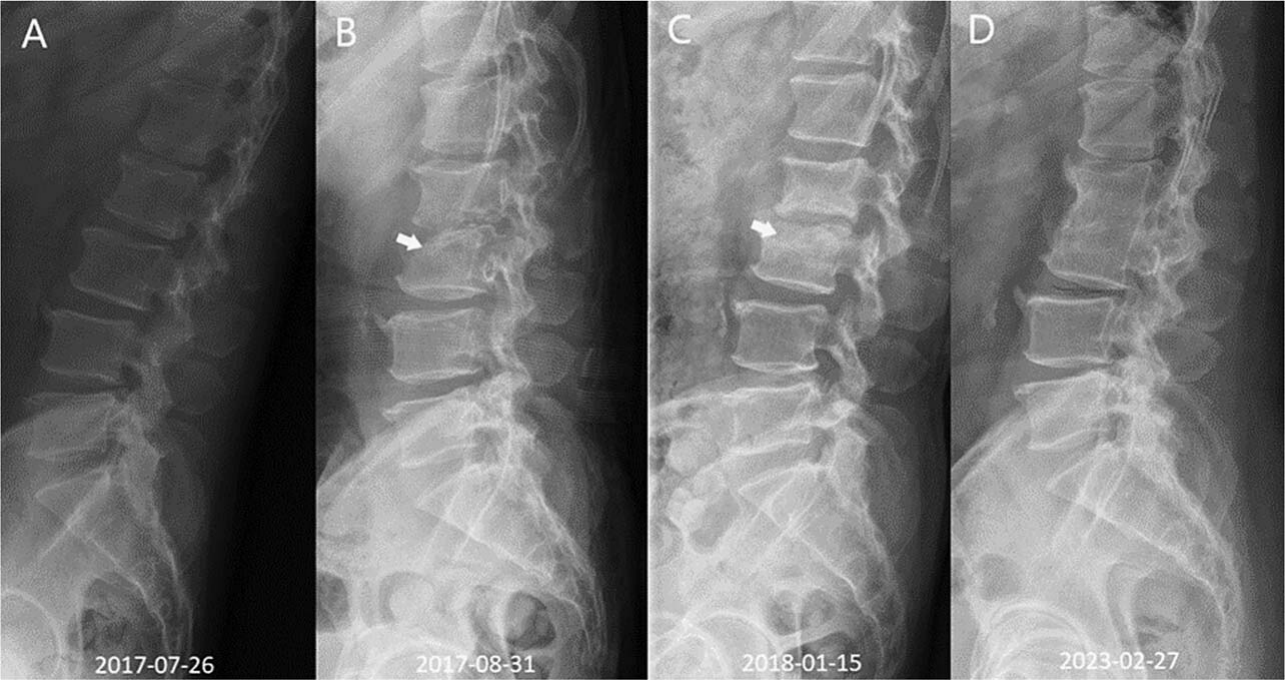
Serial lateral lumbar radiographs show progressive structural restoration after infection control and adjunctive denosumab. (A) Baseline lateral lumbar radiograph obtained on 2017-07-26, before the development of marked vertebral endplate destruction. (B) Follow-up radiograph obtained on 2017-08-31 demonstrates osteolytic destruction involving the superior endplate of L3 (arrow). (C) Follow-up radiograph obtained on 2018-01-15 shows interval re-ossification and remodeling at the affected endplate (arrow). (D) Long-term follow-up radiograph obtained on 2023-02-27 demonstrates spontaneous L2–L3 interbody fusion consistent with structural stabilization.

As the patient had declined surgical intervention, denosumab was administered adjunctively off-label 1 wk after completing antibiotic treatment during a clinically quiescent phase with the intended goal of mitigating inflammation-associated osteoclast-mediated resorption and potentially reducing the risk of progressive collapse and instability. Following treatment, the patient’s back pain improved, and he regained mobility with the aid of a Boston back brace. He received 2 60 mg s.c. denosumab injections administered 6 mo apart, after which treatment was discontinued due to significant clinical improvement. Radiographic evaluation demonstrated woven bone formation across the superior endplate of L3 following its initial endplate destruction (Figure 3). At the 6-yr follow-up, the patient remained free of adverse effects and reported sustained improvement in back pain and sciatica, with spontaneous fusion of the L2 and L3 vertebral bodies.

## Discussion

This case demonstrates radiographic vertebral remodeling and spontaneous L2-L3 interbody fusion during follow-up after infection control in a patient who later received short-course denosumab as an adjunct. Because the patient declined operative debridement and stabilization, treatment followed a nonoperative strategy centered on pathogen-directed antimicrobial therapy, immobilization with bracing, and close longitudinal follow-up. Denosumab was introduced 1 wk after antibiotic completion as an off-label, hypothesis-generating adjunct to suppress residual osteoclast-mediated bone resorption during a clinically quiescent phase. Importantly, denosumab is not a guideline-supported therapy for infectious spondylodiscitis and native vertebral osteomyelitis.

Vertebral destruction in pyogenic spondylodiscitis is not solely a consequence of direct microbial invasion. Infection-related inflammatory signaling can promote osteoclastogenesis through the RANK/RANKL axis, shifting remodeling toward resorption and accelerating structural bone loss.^3^ Experimental infection models further support a causal role of osteoclast activity, showing that RANKL-mediated osteoclast formation is required for infection-associated bone loss.^4^ In Gram-negative infections, pathogen-associated stimuli, such as lipopolysaccharide, can directly enhance osteoclast differentiation and activation and upregulate osteoclast-related genes, providing mechanistic plausibility for a persistent resorptive bias.^9^ Therefore, these observations support a biologic rationale for considering antiresorptive strategies after infection control in patients with marked endplate destruction. However, mechanistic plausibility does not establish clinical benefit, and current evidence is insufficient to recommend routine antiresorptive therapy in this setting.

Given evidence that RANKL signaling contributes to dendritic cell and T-cell function and can shape protective immune responses in infection models, initiating RANKL inhibition during active infection remains a theoretical concern for host defense.^10^^,^^11^ To address this theoretical concern, denosumab in our case was initiated only after completion of antimicrobial therapy and clinical stabilization, including defervescence and normalization of inflammatory markers. In large randomized trials of osteoporosis-dose denosumab and a dedicated post hoc safety analysis, overall serious and opportunistic infection rates were broadly similar between denosumab and placebo.^12^^,^^13^ Nevertheless, extrapolation to patients with a recent deep infection warrants caution, and careful patient selection with close monitoring remains prudent.

The clinical and radiographic improvements observed after denosumab were temporally associated and do not establish causality. Spontaneous stabilization and interbody autofusion can occur as part of the natural healing course after effective antimicrobial therapy and conservative care, and bracing and graded mobilization can also contribute to mechanical stability and remodeling.^14^ Accordingly, this report should be interpreted as hypothesis-generating rather than evidence of treatment efficacy. Baseline or follow-up BMD, bone turnover markers, and additional interval imaging beyond what is presented were not available, which further limits causal inference and contextualization of remodeling.

Safety considerations are particularly important when denosumab is used outside established indications. Medication-related osteonecrosis of the jaw is a recognized adverse effect, especially with oncology dosing; it is uncommon with the osteoporosis regimen but supports preventive dental assessment and ongoing oral surveillance.^15^^,^^16^ Rebound-associated vertebral fractures after denosumab discontinuation are another key concern, particularly when denosumab is stopped without subsequent antiresorptive therapy.^17^^,^^18^ In our patient, only 2 osteoporosis-dose injections were administered and no rebound fractures were observed over 6 yr; however, the absence of adverse events in a single case does not eliminate this risk. When denosumab is considered in similar circumstances, follow-up planning should include surveillance for new vertebral symptoms and consideration of consolidation strategies, such as bisphosphonate therapy, when clinically appropriate.^18^^,^^19^

Surgery remains indicated for selected patients who develop neurologic compromise, progressive deformity or instability, uncontrolled infection, or abscess requiring drainage. For patients who are not surgical candidates or who decline surgery, this case raises a testable hypothesis that short-course antiresorptive therapy, initiated only after unequivocal infection control, may help to limit ongoing resorption and support structural stabilization in those with marked osteolysis. Future studies should specifically evaluate whether any incremental effect exceeds expected post-infectious healing under conservative management. The outcomes of interest include recurrence, quantitative radiographic measures of stability and remodeling, patient-reported pain and function, and approaches to mitigate rebound after denosumab cessation.

## Conclusion

This case describes sustained vertebral remodeling with spontaneous L2-L3 interbody fusion following adjunctive, osteoporosis-dose denosumab administered after established clinical control of infectious spondylodiscitis. Although causality cannot be inferred from a single observation, the course supports a hypothesis that carefully timed RANKL inhibition may merit further study as a nonoperative adjunct in selected patients with pronounced infection-associated vertebral osteolysis who decline or are unsuitable for surgery. Denosumab use in this context remains off-label and non-guideline supported, and prospective data are needed to define patient selection, optimal timing, and discontinuation strategies before broader adoption.

## Data Availability

The data that support the findings of this study are not publicly available due to patient privacy concerns, but are available from the corresponding author upon reasonable request.
